# Is CT or FDG-PET more useful for evaluation of the treatment response in metastatic HER2-positive breast cancer? a case report and literature review

**DOI:** 10.3389/fonc.2023.1158797

**Published:** 2023-04-20

**Authors:** Hirotaka Suto, Yumiko Inui, Atsuo Okamura

**Affiliations:** ^1^Department of Medical Oncology, The Cancer Institute Hospital of Japanese Foundation for Cancer Research, Tokyo, Japan; ^2^Department of Medical Oncology/Hematology, Kakogawa Central City Hospital, Hyogo, Japan

**Keywords:** breast cancer, HER2, liver metastasis, CT, FDG-PET, RECIST

## Abstract

Response evaluation criteria in solid tumors version 1.1 (RECIST ver1.1) has been widely adopted to evaluate treatment efficacy in solid tumors, including breast cancer (BC), in clinical trials and clinical practice. RECIST is based mainly on computed tomography (CT) images, and the role of fluorodeoxyglucose-positron emission tomography (FDG-PET) is limited. However, because the rate of tumor shrinkage on CT does not necessarily reflect the potential remaining tumor cells, there may be a discrepancy between the treatment response and prognosis in some cases. Here we report a case of metastatic human epidermal growth factor receptor 2 (HER2)-positive BC where FDG-PET was preferable to CT for evaluation of the treatment response. A 40-year-old woman became aware of a lump in her right breast in September 201X. She was pregnant and underwent further examinations, including a biopsy, in November. The diagnosis was HER2-positive BC (cT2N2bM1, stage IV). Trastuzumab plus pertuzumab plus docetaxel (TPD) therapy was initiated in December 201X. CT performed in February 201X+1 showed cystic changes in the metastatic lesions in the liver, and the treatment response was stable disease (SD) according to RECIST. However, FDG-PET in March 201X+1 did not detect abnormal uptake of FDG in the hepatic lesions. The disease remained stable thereafter. Thus, tumor shrinkage may not be apparent in situations where the response to treatment results in rapid changes in blood flow within the tumor, which is associated with cystic changes. When patients with hypervascular liver metastases receive treatment with highly effective regimens, the target lesion may show cystic changes rather than shrinkage, as observed in the present case. Therefore, FDG-PET is sometimes superior to CT in judging a tumor response.

## 1 Introduction

Response evaluation criteria in solid tumors version 1.1 (RECIST ver1.1) has been widely adopted to evaluate treatment efficacy in solid tumors, including breast cancer (BC), in clinical trials and clinical practice (1). RECIST ver1.1 is mainly based on computed tomography (CT) images and is useful for the evaluation of cytotoxic anticancer therapy as well as molecular-targeted drug therapy (2). The role of ^18^F-fluorodeoxyglucose-positron emission tomography (FDG-PET) in the determination of the treatment efficacy is limited. However, because tumor shrinkage based on CT images does not always correspond to tumor cell residuals, scattered cases have been reported in which the treatment efficacy determination and prognosis are divergent (3–7). Conversely, FDG-PET can evaluate tumor activity by glucose uptake. Hence, in Europe and the United States, quantitative treatment response determination by FDG-PET has been attempted, with the recommendation of FDG-PET by the European organization for research and treatment of cancer (8) and the PET Response Criteria in Solid Tumors (9). Although several studies have used FDG-PET to determine the efficacy of neoadjuvant chemotherapy against human epidermal growth factor receptor 2 (HER2)-positive BC (10–13), few studies have examined the utility of FDG-PET in determining the efficacy of treatment for metastatic HER2-positive BC (14). Here we report a case of metastatic HER2-positive BC where FDG-PET was preferable to CT for evaluation of the treatment response.

## 2 Case report

**Figure 1:**
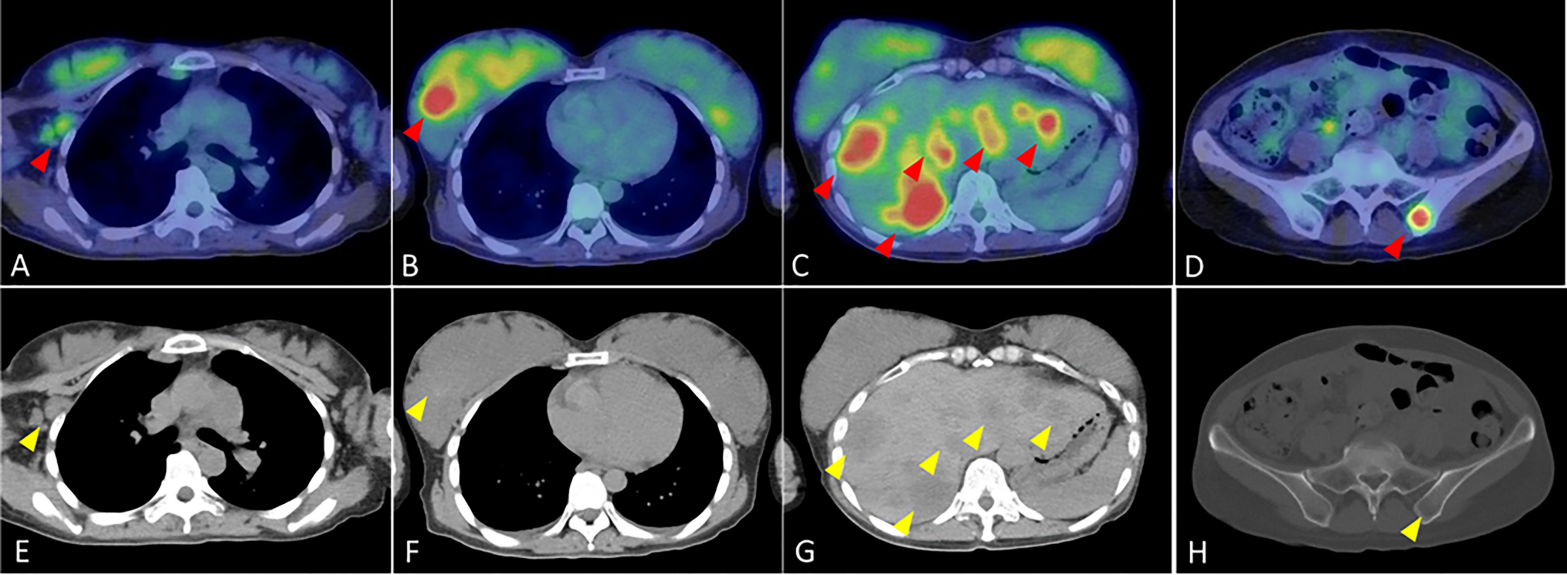
Fluorodeoxyglucose-positron emission tomography/computed tomography findings at the initial visit to our department for the patient with human epidermal growth factor receptor 2-positive breast cancer. **(A)** High FDG accumulation in the level I–II region of the right axilla (red arrow) **(B)** High FDG accumulation in the upper outer quadrant of the right breast (red arrow) **(C)** Numerous foci of high FDG accumulation in the liver (red arrows). **(D)** High FDG accumulation in the left iliac bone (red arrow). **(E)** Enlarged lymph node in the level I-II region of the right axilla (yellow arrow). **(F)** Mass in the upper outer right breast (yellow arrow). **(G)** Multiple low density areas in the liver (yellow arrows). **(H)** Low density area in pelvic region (yellow arrow).

**Figure 2:**
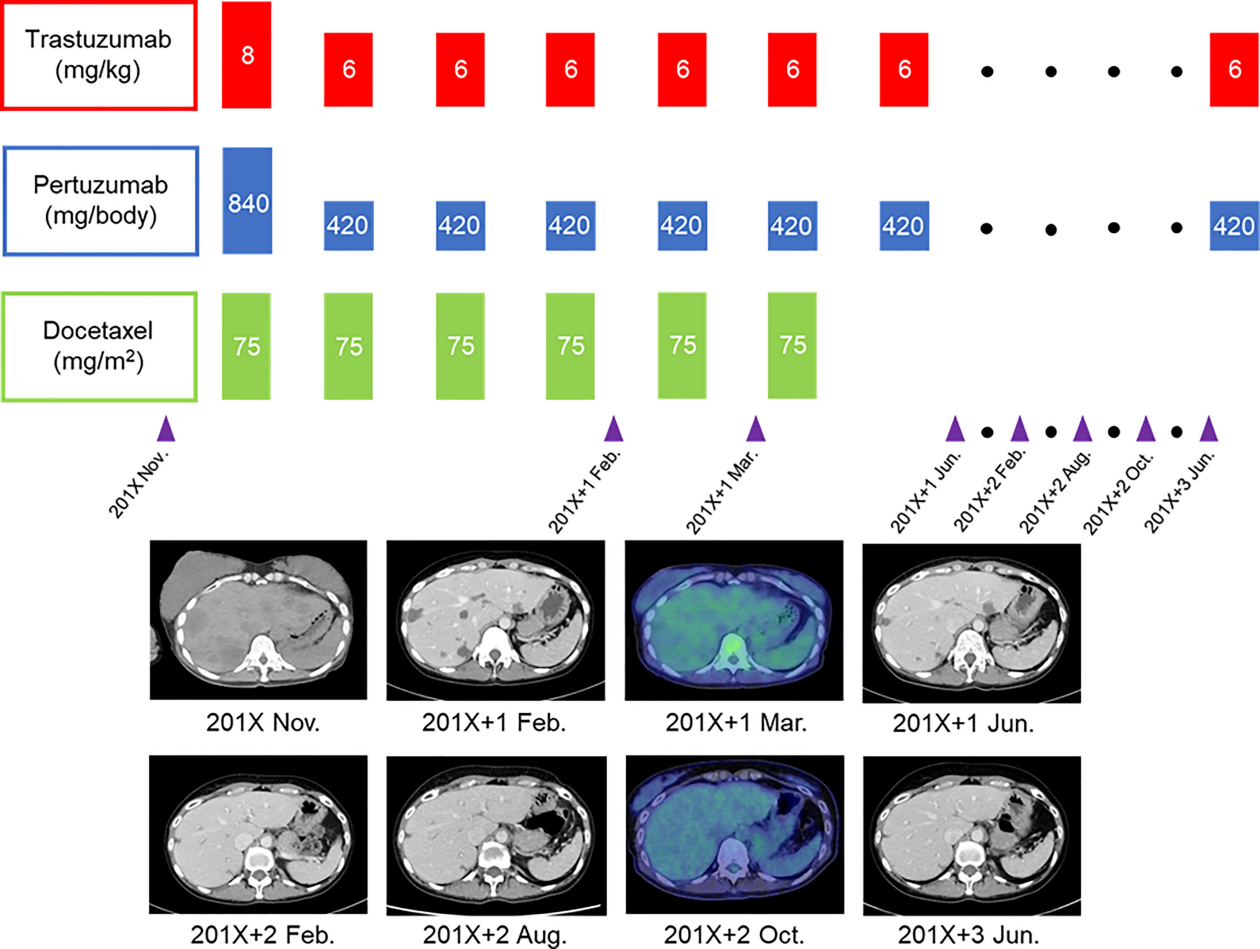
Course of treatment and imaging changes in multiple liver metastases for the patient with human epidermal growth factor receptor 2-positive breast cancer.

A 40-year-old woman became aware of a lump in her right breast in September 201X. Because she was pregnant, she underwent a cesarean section in mid-November and underwent further examinations, including a core needle biopsy, in late November. Physical examination at the initial visit to our department revealed a body temperature of 36.5°C; a heart rate of 78 beats/min; blood pressure of 122/74 mmHg; a respiratory rate of 12 breaths/minute; no eyelid conjunctiva pallor; no heart murmur; flat, soft, non-tender abdomen; no edema; a palpable, 2-cm, elastic, firm mass in the upper outer quadrant of the right breast; and palpable and swollen right axillary lymph nodes. Breast ultrasound revealed a hypoechoic mass measuring 32.6 × 16.2 mm and showing well-defined borders and a heterogeneous interior in the upper outer quadrant of the right breast. Blood tests showed mildly elevated liver enzymes, high serum alkaline phosphatase and serum lactate dehydrogenase (LDH) levels, and markedly elevated carcinoembryonic antigen (CEA) and carbohydrate antigen 15-3 (CA15-3) levels (**Table 1**). FDG-PET/CT revealed high FDG accumulation in the upper outer quadrant of the right breast (standardized uptake value (SUV) max, 7.519), enlarged lymph nodes, and high FDG accumulation in the level I–II region of the right axilla and internal mammary lymph node region (SUV max, 3.525), numerous low-density areas with high FDG accumulation in the liver (SUV max, 7.816), and high FDG accumulation in the left iliac bone (SUV max, 7.356) (**Figure 1**). The histopathological diagnosis based on core needle biopsy from the breast mass was invasive ductal carcinoma of the breast (estrogen receptor (ER)-positive, progesterone receptor-negative, HER2 3+, Ki-67 40%). The clinical stage by imaging was cT2N2bM1[OSS, HEP], stage IV. Trastuzumab plus pertuzumab plus docetaxel (TPD) therapy for metastatic HER2-positive BC was initiated in December 201X. Blood tests on the day after treatment showed the following: aspartate aminotransferase (AST), 341 IU/L; alanine aminotransferase (ALT), 155 IU/L; LDH, 4021 IU/L; and liver dysfunction. However, there were no findings indicating suspected tumor lysis syndrome, with a serum creatinine level of 0.48 mg/dL, uric acid level of 5.2 mg/dL, potassium level of 3.9 mmol/L, and phosphorus level of 3.6 mg/dL. Blood tests performed 2 days after the start of chemotherapy showed the following: AST, 187 IU/L; ALT, 143 IU/L; and LDH, 2151 IU/L, with liver dysfunction and LDH levels also showing an improvement trend. At the start of the second course of treatment, the patient’s liver enzymes were within normal limits, and she continued treatment. In February 201X+1, the CEA and CA15-3 levels were 90.2 ng/mL and 33.0 IU/mL, respectively. CT performed in the same period showed cystic changes in the metastatic lesions in the liver, and the treatment response was stable disease according to RECIST (**Figure 2**). However, FDG-PET performed in March 201X+1 did not detect abnormal uptake of FDG in the hepatic lesions (**Figure 2**; **Supplementary Figure 1**). CT performed in June 201X+1 showed shrinkage of the liver metastases, and the disease remained stable for more than three years (**Figure 2**).

**Table 1:** Laboratory data obtained at the initial visit to our department for a patient with human epidermal growth factor receptor 2-positive breast cancer.

Blood components	Patient		Normal range
**Complete blood count**			
**White blood cells**	7340	/μL	3300–8600
**Red blood cells**	466x10^4^	/μL	386-492 × 10^4^
**Hemoglobin**	14.1	g/dL	11.6–14.8
**Hematocrit**	43.9	%	35.1–44.4
**Mean corpuscular volume**	94	fL	83.6–98.2
**Platelets**	30.9x10^4^	/μL	158-348 × 10^4^
**Neutrophils**	79	%	40.0–70.0
**Lymphocytes**	10	%	20.0–50.0
**Monocytes**	6	%	0.0–10.0
**Eosinocytes**	2	%	1.0–5.0
**Basocytes**	1	%	0.0–1.0
**Biochemistry**			
**Total protein**	6.8	g/dL	6.6–8.1
**Albumin**	3.7	g/dL	4.1–5.1
**C-reactive protein**	0.27	mg/dL	0.00–0.14
**Aspartate aminotransferase**	55	IU/L	13–30
**Alanine aminotransferase**	48	IU/L	7–23
**Alkaline phosphatase**	925	IU/L	106–322
**Total bilirubin**	0.7	mg/dL	0.4–1.5
**Lactate dehydrogenase**	605	IU/L	124–222
**Blood urea nitrogen**	13.1	mg/dL	8.0–20.0
**Creatinine**	0.46	mg/dL	0.46–0.79
**Uric acid**	4.8	mg/dL	2.6–5.5
**Na**	142	mEq/L	138–145
**K**	3.7	mEq/L	3.6–4.8
**Cl**	104	mEq/L	101–108
**Ca**	9.4	mg/dL	8.8–10.1
**P**	2.9	mg/dL	2.7–4.6
**Creatine kinase**	78	IU/L	41–153
**Amylase**	75	IU/L	44–132
**Glucose**	152	mg/dL	73–109
**CEA**	2365	ng/mL	0.0–5.0
**CA15-3**	154	IU/mL	0.0–37.0

Na, sodium; K, potassium; Cl, chlorine; Ca, calcium; P, phosphorus; CEA, carcinoembryonic antigen; CA15-3, carbohydrate antigen 15-3.

## 3 Discussion

We presented a case of HER2-positive BC with liver metastasis where FDG-PET was valuable for the assessment of the therapeutic response. The patient, who showed an early response according to FDG-PET, continued to respond to treatment three years after the start of treatment.

In some reports, the pathological complete response rate after neoadjuvant chemotherapy for HER2-positive BC has correlated with the treatment response evaluated by FDG-PET (10–13, 15–21), whereas no correlation has been observed in other studies (22–26). Furthermore, for BC, the utility of FDG-PET may differ between primary sites and metastatic lymph nodes (27). Furthermore, the ability of PET to detect breast cancer is highly dependent on tumor size: the sensitivity for tumors less than 1 cm in diameter was 25%, whereas the sensitivity for tumors between 1 cm and 2 cm in diameter was 84.4% (28). On the other hand, RECIST ver1.1, based on CT imaging, reportedly shows efficacy in determining the therapeutic effect of molecular-targeted drug therapy (2). Therefore, the routine use of FDG-PET for determining the treatment response in BC is not recommended.

However, HER2/ER-positive breast cancer may be the most suitable breast cancer subtype for FDG-PET. The rationale for their suitability is that glucose transporters (GLUT) on cell membranes and cell proliferative capacity influence FDG accumulation (29). The Phosphatidylinositol 3-kinase (PI3K)/Akt/mammalian target of rapamycin (mTOR) pathway is also involved in the expression and function of GLUTs, which are involved in glucose uptake (30). HER2/ER-positive breast cancer often has high Ki67 levels, a marker of cell proliferative potential, and the PI3K/Akt/mTOR pathway is also activated (31). If treatment for this breast cancer subtype is successful, a decrease in FDG accumulation may be detected earlier than morphological shrinkage by CT because of the expected reduced expression of GLUT and Ki67 values. Furthermore, there are reports that FDG-PET affects the prognosis of breast cancer patients (32, 33). That is because FDG-PET has a high diagnostic ability for distant metastasis, especially in breast cancer patients with bone metastasis (34, 35). Therefore, FDG-PET may be useful not only for detecting distant metastases that are difficult to detect with CT in staging but also for follow-up.

In addition, FDG-PET is useful for determining the response to drug treatment in patients with gastrointestinal stromal tumors (GISTs) (3, 4, 36–38). Therefore, FDG-PET is preferred over RECIST for evaluation of the response to treatment (39). The characteristics of GISTs and their treatment include the presence of hypervascular liver metastases (40–42) and a high response rate to imatinib therapy (43). Approximately two-thirds of GISTs have *KIT* exon11 mutations (40, 44). The response rate for imatinib in patients with untreated metastatic GISTs with *KIT* exon11 mutations reportedly ranges from 68% to 72% (45–47) (**Table 2**). High-response chemotherapy for hypervascular tumors leads to rapid blood flow changes. This can result in internal necrosis and cystic transformation without tumor shrinkage, which may occur during the treatment of GISTs (55). In such cases, FDG-PET is more suitable for determining the treatment response than RECIST.

**Table 2:** Reported response rates for chemotherapy according to the cancer type.

Cancer type	Subtype	Phase	Setting	Regimen	ORR
BC (48)	HER2-positive	II	NAC	TPD	88.00%
BC (49)	HER2-positive	III	NAC	TPD	88.60%
BC (50)	HER2-positive	III	Palliative	TPD	80.20%
GIST (45)	*KIT* exon11 mutant	III	Palliative	Imatinib	67.70%
GIST (46)	*KIT* exon11 mutant	III	Palliative	Imatinib	71.70%
GIST (47)	*KIT* exon11 mutant	III	Palliative	Imatinib	68.80%
CRC (51)	All comer	II	LM only	FOLFOXIRI+Bev	80.50%
CRC (52)	All comer	II	LM only	FOLFOXIRI+Bev or C-mab	75.00%
CRC (53)	*RAS/BRAF* wild	II	LM only	FOLFOXIRI+C-mab	95.50%
CRC (54)	*KRAS* wild	II	LM only	FOLFOXIRI+P-mab	60.00%

ORR, overall response rate; BC, breast cancer; NAC, neoadjuvant chemotherapy; TPD, trastuzumab plus pertuzumab plus docetaxel; GIST, gastrointestinal stromal tumor; CRC, colorectal cancer; LM, liver metastasis; FOLFOXIRI+Bev, 5-fluorouracil/leucovorin/oxaliplatin/irinotecan plus bevacizumab; C-mab, cetuximab; P-mab, panitumumab.

The response rate for the TPD regimen used for untreated HER2-positive BC reportedly ranges from 80.2% to 88.6% (48–50) (**Table 2**), and some cases of hepatic metastases from BC show hypervascular patterns (56, 57). In addition, the response rate for triplet plus bevacizumab or anti-epidermal growth factor receptor antibody treatment in patients with untreated colorectal cancer (CRC) with liver metastases ranges from 60.0% to 95.5% (51–54) (**Table 2**). However, liver metastases from CRC are generally hypovascular tumors (55). Therefore, they are less frequently cystic, similar to GISTs. Meanwhile, when angiogenesis inhibitors are administered, the tumor blood flow is rapidly altered and the liver metastases from CRC may become cystic; this suggests that RECIST is inappropriate for determining the treatment efficacy (58).

The present case involved untreated HER2-positive BC with liver metastases, and the LDH levels after initiation of the TPD regimen suggested a high response within a few days. Patients with such a significant reaction to hypervascular liver metastases within a few days are prone to cystic transformation of the liver metastases.

In summary, when liver metastases do not shrink and become cystic despite a high response to chemotherapy, FDG-PET may be more suitable than CT-based RECIST for determination of the treatment response.

## Data availability statement

The original contributions presented in the study are included in the article/**Supplementary Material**. Further inquiries can be directed to the corresponding author.

## Ethics statement

Ethical review and approval was not required for the study on human participants in accordance with the local legislation and institutional requirements. The patient provided written informed consent to participate in this study. Written informed consent was obtained from the patient for the publication of this case report.

## Author contributions

Conceptualization, HS and AO. Methodology, HS and AO. Investigation, HS, YI, and AO. Data curation, HS, YI, and AO. Writing—original draft preparation, HS. Writing—review and editing, HS, YI, and AO. Supervision, AO. All authors have read and agreed to the published version of the manuscript.
