# Supplementary material for: Is CT or FDG-PET more useful for evaluation of the treatment response in metastatic HER2-positive breast cancer? a case report and literature review
Source: Front Oncol. 2023 Apr 20;13:1158797. doi: 10.3389/fonc.2023.1158797 (PMC10157226; doi:10.3389/fonc.2023.1158797)
Supplement: Supplementary file 1 [file DataSheet_1.docx]

Supplementary Material

Is CT or FDG-PET More Useful for Evaluation of the Treatment Response in Metastatic HER2-positive Breast Cancer? A Case Report and Literature Review.

Hirotaka Suto^1,2*^, Yumiko Inui^2^, Atsuo Okamura^2^

*** Correspondence:** Hirotaka Suto: [hirotaka.suto@jfcr.or.jp](mailto:hirotaka.suto@jfcr.or.jp)

# Supplementary Figures


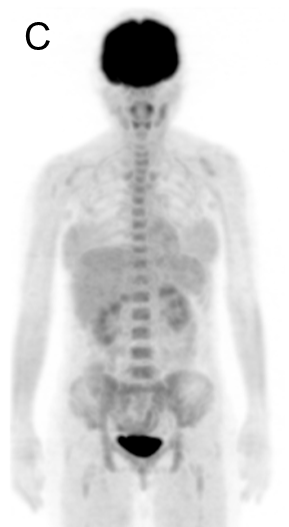

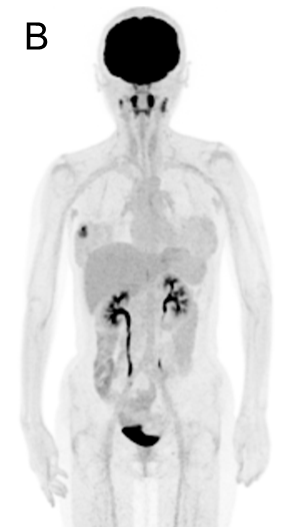

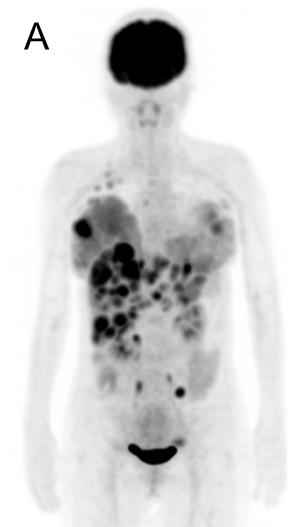


A. MIP image on November, 201X B. MIP image on March, 201X+1 C. MIP image on October, 201X+2

**Supplementary Figure 1.** Maximun Intensity Projection (MIP) images of positron emission tomography for the patient with human epidermal growth factor receptor 2-positive breast cancer
